# Authors' reply

**DOI:** 10.4103/0301-4738.53070

**Published:** 2009

**Authors:** Nazimul Hussain, Subhadra Jalali, Alka Rani, Hema Rawal

**Affiliations:** Smt. Kanuri Santhamma Retina Vitreous Centre, LV Prasad Eye Institute, Hyderabad, India

Sir,

We want to thank the reader[1] for taking keen interest in this case report which depicts the most adverse effect of ICG on retinal pigment epithelium (RPE) cells.[2]

We have clearly mentioned in the article that while fluid-air exchange was performed using silicone brush cannula, the macula flattened. This may also suggest that microhole was present in the foveal area which was also evident when ICG migrated subretinally. Hence, a retinotomy was not created. We used silicone brush or silicone-tipped flute needle for all macular surgery. We appreciate author's conclusion.

The choice of intraocular tamponade was not based on the occurrence of macular pucker as mentioned by authors.[3] In fact, the choice would have been intraocular gas either SF6 or C3F8. However, our decision was based on the following: 1. Extensive area of RPE ICG staining which may affect the re-attachment of the macula as a large area of serous detachment was seen preoperatively on OCT and clinically.[3] 2. Patient did not want gas tamponade as discussed preoperatively. She had surgery earlier for vitreomacular traction with serous macular detachment with intraocular gas.

We appreciate author's concerns regarding the management of the case.
